# Biotransformation of lignan glycoside to its aglycone by *Woodfordia fruticosa* flowers: quantification of compounds using a validated HPTLC method

**DOI:** 10.1080/13880209.2016.1238948

**Published:** 2016-12-08

**Authors:** Shikha Mishra, Vidhu Aeri

**Affiliations:** Department of Pharmacognosy and Phytochemistry, Jamia Hamdard, New Delhi, India

**Keywords:** Lyoniside, lyoniresinol, validation, Asokarista^®^, NMR

## Abstract

**Context:***Saraca asoca* Linn. (Caesalpiniaceae) is an important traditional remedy for gynaecological disorders and it contains lyoniside, an aryl tetralin lignan glycoside. The aglycone of lyoniside, lyoniresinol possesses structural similarity to enterolignan precursors which are established phytoestrogens.

**Objectives:** This work illustrates biotransformation of lyoniside to lyoniresinol using *Woodfordia fruticosa* Kurz. (Lythraceae) flowers and simultaneous quantification of lyoniside and lyoniresinol using a validated HPTLC method.

**Materials and methods:** The aqueous extract prepared from *S. asoca* bark was fermented using *W. fruticosa* flowers. The substrate and fermented product both were simultaneously analyzed using solvent system:toluene:ethyl acetate:formic acid (4:3:0.4) at 254 nm. The method was validated for specificity, accuracy, precision, linearity, sensitivity and robustness as per ICH guidelines.

**Results:** The substrate showed the presence of lyoniside, however, it decreased as the fermentation proceeded. On 3rd day, lyoniresinol starts appearing in the medium. In 8 days duration most of the lyoniside converted to lyoniresinol. The developed method was specific for lyoniside and lyoniresinol. Lyoniside and lyoniresinol showed linearity in the range of 250–3000 and 500–2500 ng. The method was accurate as resulted in 99.84% and 99.83% recovery, respectively, for lyoniside and lyoniresinol.

**Conclusion:** Aryl tetralin lignan glycoside, lyoniside was successfully transformed into lyoniresinol using *W. fruticosa* flowers and their contents were simultaneously analyzed using developed validated HPTLC method.

## Introduction

**Figure 1.**
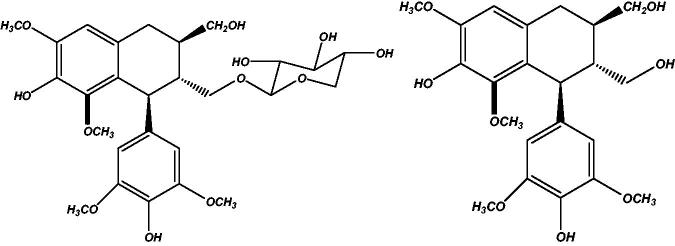
Structures of lyoniside (**1**) and lyoniresinol (**2**).

Lignans are poly-phenolic compounds structurally described by 8-8′ coupling of two phenylpropanoid units. They have their roles in fight against phytopathogenic organisms, protection against stress and growth regulation. In mammals, metabolites of plant lignans, enterolignans, have been reported to possess antiestrogenic, weakly estrogenic, anticarcinogenic, antioxidant, antimicrobial effects (Schwartz & Sontag 2006). Lyoniside (**1**) is a xyloside of lyoniresinol (**2**) (Figure 1) which is an aryltetralin lignan (Dada et al. 1989). Several activities have been reported for lyoniside as well as lyoniresinol. Lyoniside has been reported to possess antifungal (Szakiel et al. 2011) and topoisomerase inhibitory activity (Mukherjee et al. 2012) whereas lyoniresinol exhibited antimelanogenic (Liu et al. 2013) and antitrichomonal activity (Moo-PucI et al. 2014). Aglycones are more lipophilic compounds than its parent glycosides.

This study presents a simple, sustainable method for biotransformation of glycoside into its aglycone along with development and validation of a simultaneous HPTLC-densitometric analysis method.

## Materials and methods

### Plant material

*Saraca asoca* Linn. (Caesalpiniaceae) bark was procured from a commercial supplier (Global herbs, Khari Baoli, New Delhi) in March 2011 and authenticated by Dr. H.B Singh, RHMD, NISCAIR, Delhi. A voucher specimen (PRL/JH/11/11) was kept in Department of Pharmacognosy & Phytochemistry, Jamia Hamdard, New Delhi, India. All other chemicals were from SD Fine Chemicals Pvt Ltd, New Delhi, India.

### Reference standards

Lyoniside and lyoniresinol were purchased from Chemfaces Pvt. Ltd, Wuhan, People’s Republic of China.

### Instruments

For determination of UV maxima the sample solution was scanned between the wavelength range of 400 nm to 200 nm using the Shimadzu double beam UV visible spectrophotometer Shimadzu UV – 1601 (Shimadzu Corp, Kyoto, Japan) in the spectrum mode.

For mass analysis, the sample was directly injected in mass spectrometer. Agilent LCMS (Model:6410B) was used with RP-LC Column: C18, 50 mm × 2.1 mm, 1.8 μm particle size and maintained at 40 °C. MS detection was achieved using an electrospray ion (ESI) source in positive mode. The ionization source of the MS detector had 4.0 kV capillary voltage, 350 °C source temperature and 9 L/min gas flow rate (both gases were nitrogen). Data were acquired and processed using the Mass hunter software.

For NMR analysis, Bruker Avance DRX 500 (^1^H at 500 MHz, Canton, Massachusetts, USA). The spectra were acquired in CD_3_OD at 293 K.

### Preparation of extracts

*Saraca asoca* bark was powdered and sieved through sieve no 44. Powdered material (300 g) was boiled with 3000 mL distilled water until the volume was reduced to 1/4th. It was then vacuum filtered and stored at 4 °C until further use.

### Biotransformation

*Woodfordia fruticosa* Kurz. (Lythraceae) flowers were washed with sterile water and dried. The extract (25 mL) was poured into 100 mL Erlenmayer flasks and each flask is added with *W. fruticosa* flowers (1.6 g). The flasks were closed by cotton plug. All the flasks were kept in incubator at 28 ± 2 °C. Each subsequent day, samples were taken out and analyzed for the content of lyoniside and lyoniresinol using HPTLC.

### Preparation of standards

Reference standard compound (5 mg) was dissolved in 5 mL methanol to obtain a stock standard solution of 1 mg/mL.

### Chromatographic conditions

CAMAG HPTLC system (Switzerland) equipped with a Linomat 5 sample applicator was used for HPTLC analysis. Chromatography was done on aluminum backed HPTLC plates precoated with silica gel 60F254 (20 cm × 20 cm, 0.2 mm thickness, 2–3 μm particle size (Merck, Dermastadt, Germany). TLC plates were pre-washed with methanol and dried at 110 °C for 45 minutes for activation. Variable volumes of the standard solutions were spotted as bands of 5 mm width by using the auto sampler fitted with a 100 μL Hamilton syringe. The plates were developed using various combinations of toluene, ethyl acetate and formic acid in a CAMAG twin-trough chamber, presaturated with 25 mL mobile phase. The developed plates were air dried and scanned at 254 nm. Spots of lyoniside and lyoniresinol were scanned from 200 to 400 nm so as to record their UV spectrum. Densitograms were recorded at the 254 nm.

### Method validation

The developed method was validated as per the ICH guidelines. The following parameters were assessed for validating the method.

Sensitivity was determined in terms of LOD and LOQ and signal to noise ratio method was followed for their determination. Further, linearity was established for both the reference standards. Graded concentration of reference standards were applied onto the plate, developed and scanned. Plots were constructed between concentration of reference standards and corresponding peak area to obtain a linearity profile for each compound (Koll et al. 2003).

The specificity of the method was determined by determining peak purity for individual markers. The UV spectra of respective bands were compared in reference standard and marketed formulation/extract. Further, the respective bands were scrapped and dissolved in methanol. It was then filtered and injected into MS interface. For NMR analysis, the methanol solution of scrapped band was dried and taken into deuterated chloroform. Precision of the method was determined in terms of intra- and inter-day variation studies. Lyoniside and lyoniresinol (500–1500 ng/band) were evaluated on the same day and after three days. The % RSD was taken as a mean of precision. Pre-analyzed samples were added with known quantity (50, 100, 150%) of reference standards (500 ng/band). The percentage recovery was estimated. The robustness of developed method was determined by evaluating the effect of small, planned variation of the analytical conditions on the peak areas of the markers. Factors analyzed were mobile phase composition (0.5 mL) and detection wavelength (4 nm). The standard deviation of peak areas and % relative standard deviation (%RSD) were calculated for each variable factor. The stability of reference standard solutions (500 ng/band) was determined by comparing the peak area in respective chromatograms developed at 0 and 24 h of storage at room temperature (Koll et al. 2003).

### Data and statistical analysis

A spectro-densitometer (Scanner 3, CAMAG) equipped with ‘WINCATS’ planar chromatography manager (version 1.3.0) software was used for the densitometry measurements, spectra recording and data processing. Each concentration of the reference compound was spotted in triplicate on the plates and analyzed. The concentrations of reference compounds were plotted against peak area to obtain calibration curve.

## Results and discussion

The present work was inspired by a well-known ayurvedic formulation, Asokarista. This polyherbal formation is known as a menstrual cycle regulator. The major component of this formulation is *Saraca asoca* which contain lyoniside as major chemical constituent, however, this compound has not been reported for any gynaecological activity. The formulation is a fermented product so authors hypothesized that lyoniside gets converted to some other compound during fermentation which might have gynaecological properties. Biotransformation is a process which can be defined as the use of biological system to induce chemical changes in compounds that are not their natural substrates.

The present work was done to estimate the content of lyoniside and lyoniresinol in aqueous extract and fermented extract over the duration of fermentation (8 days). Also, a simple and specific HPTLC-densitometric method was developed and validated in terms of accuracy, precision, sensitivity, robustness parameters for simultaneously determining their content.

### Analysis of the samples

**Figure 2.**
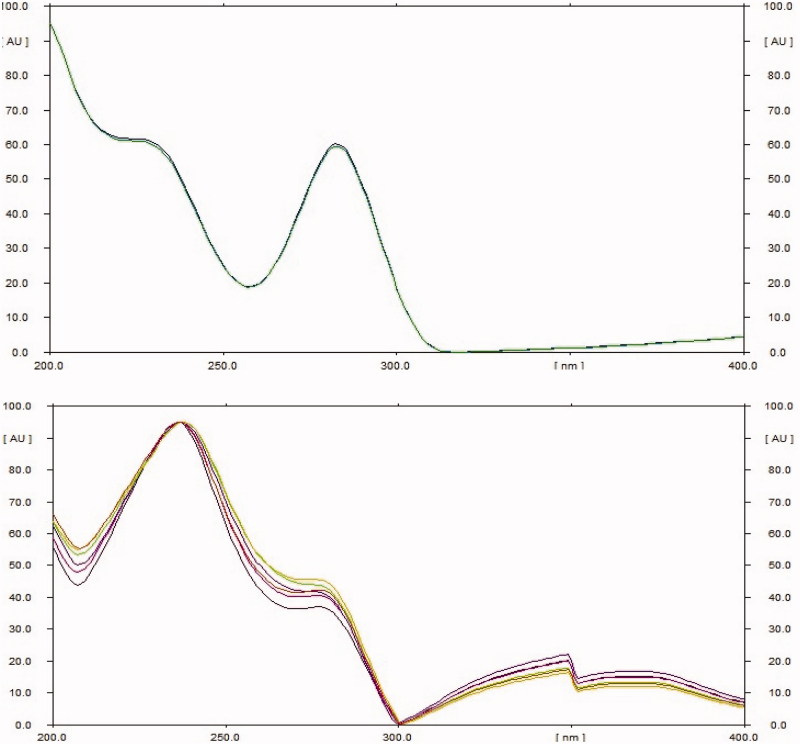
UV spectra for lyoniside and lyoniresinol.

**Figure 3.**
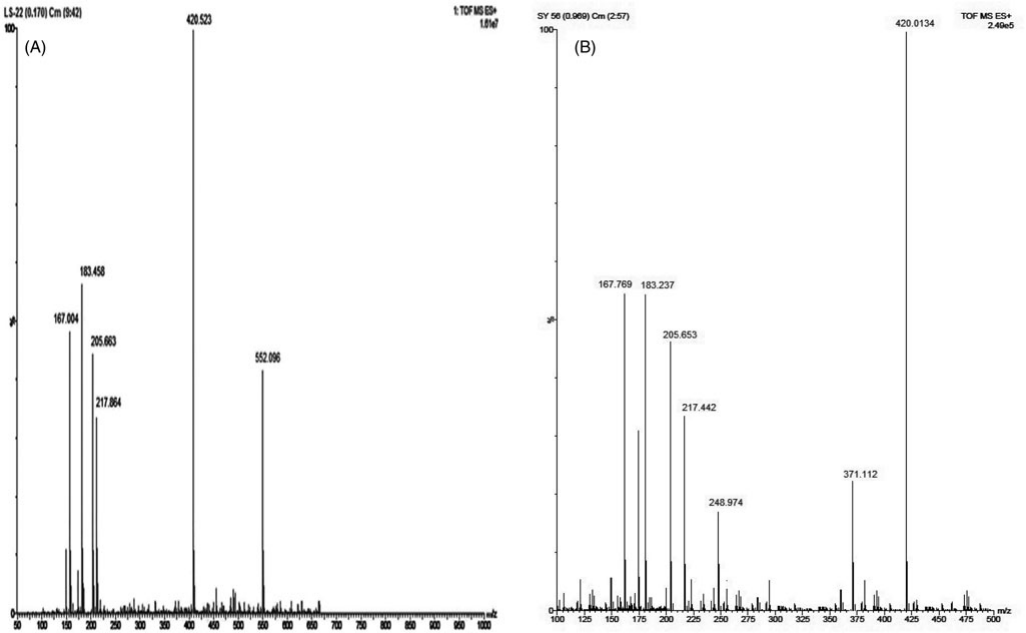
(a) Mass spectra for lyoniside. (b) Mass spectra for lyoniresinol.

**Figure 4.**
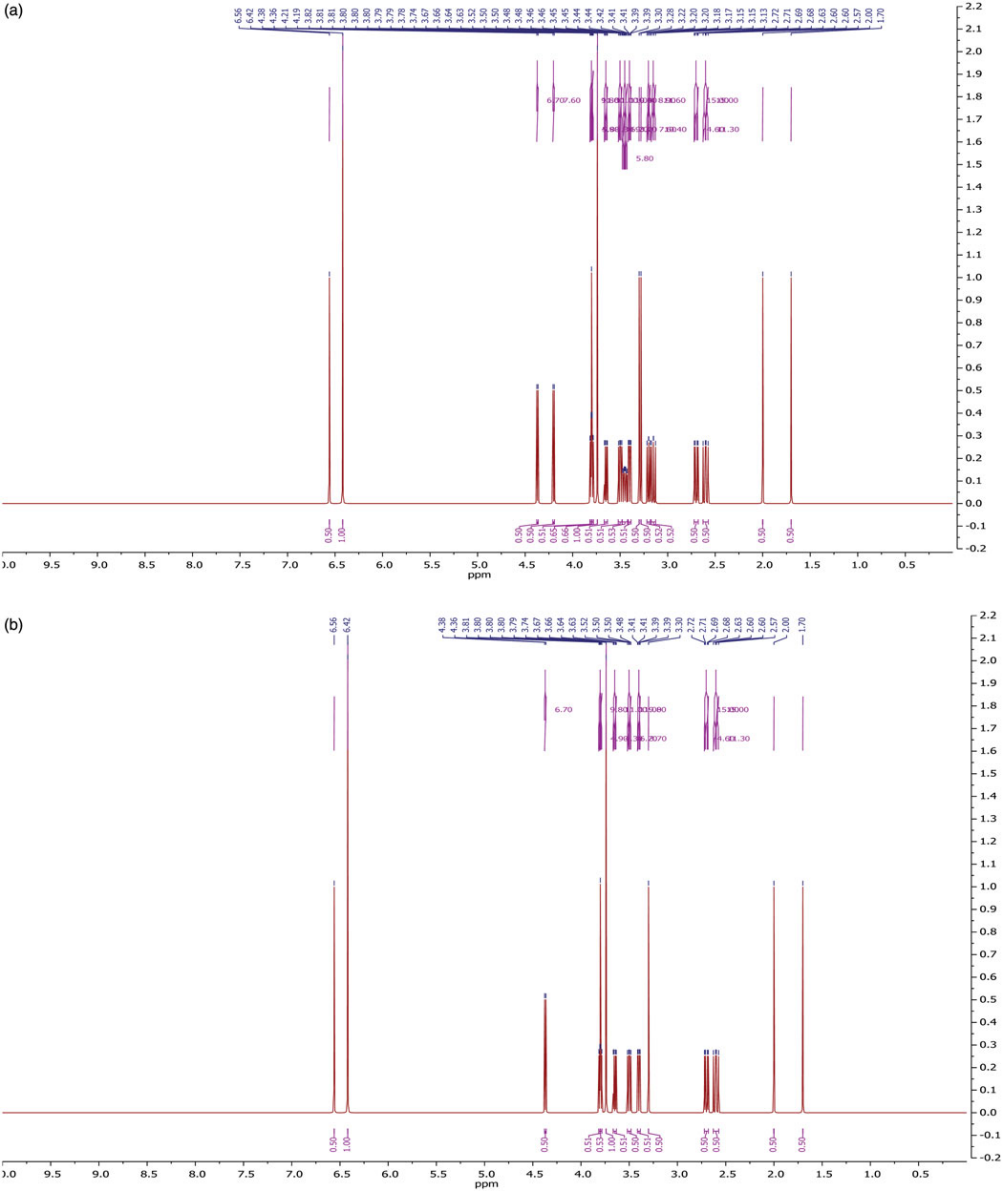
(a) NMR spectra for lyoniside. (b) NMR spectra for lyoniresinol.

The extract was applied in triplicate on TLC plate and developed in optimized solvent system. On day 0, a peak at 0.31 R_f_ was prominent. This peak had UV maxima at 283 nm (Figure 2). Also its mass spectrum showed peaks at 552 (M^+^) and 420 (base peak). Other fragments were 217 (33%), 205 (45%), 183 (55%) and 167 (49%) (Figure 3). Its NMR spectra showed an anomeric proton at 4.3 ppm indicating it to be a glycoside (Figure 4), identified as lyoniside when compared with literature values (UV, MS and NMR) (Sadhu et al. 2007). It indicates that this glycoside is not hydrolyzed during boiling with water over an extended period of time.

### Biotransformation

*Woodfordia fruticosa* flowers are famed for being the traditional inoculum in Ayurveda, the Indian traditional medicinal system which is more than 2000 years old. It has been reported that these flowers contain microbes. The visible colony formation starts from 2^nd^ day. Simultaneously, the HPTLC profile starts changing. A peak starts rising at 0.9 R_f_ (Figure 2). This peak had UV maxima at 237 nm (Figure 2). Also its mass spectrum showed peak at 420 (M^+^, base peak). Further fragmentation showed m/z 371(21%), 248 (18%), 217 (33%), 205 (43%), 183 (64%) and 167 (63%) (Figure 3) Its NMR spectra showed no additional NMR signals indicating it to be an aglycone (Figure 4). When compared with literature values (UV, MS and NMR), it was identified as lyoniresinol, the aglycone of lyoniside. The identity of sugar was determined by Bials’ test and osazone formation. The sugar was confirmed to be xylose.

Another, interesting observation was generation of ethanol in the biotransformation product.

### Method development

Development of a suitable solvent system is crucial for simultaneous estimation of compounds. The challenge here was to simultaneously record the peaks for an aglycone and its glycoside with suitable R_f_. Various combinations were checked and toluene:ethyl acetate:formic acid (4:3:0.4) solvent system was found suitable as the peaks of lyoniside and lyoniresinol were resolved at 0.3 and 0.9, respectively.

### Method validation

Linearity was checked by constructing a five point calibration curve between peak area and concentrations. Linear profiles were generated for lyoniside and lyoniresinol at 250–3000 ng and 500–2500 ng, respectively. A good linear relation was observed with *r*^2^ value of 0.9996 and 0.9988 for lyoniside and lyoniresinol, respectively (Table 1).

**Table 1.** Validation parameters for lyoniside and lyoniresinol.

Validation parameter	Lyoniside	Lyoniresinol
Linearity range (ng spot^−1^)	250–3000	500–2500
Correlation coefficient (*r*^2^)	0.9996; 0.9997	0.9988; 0.9994
Regression equation	6.9228 × −233.26	6.938 × −264.004
Limit of detection (ng spot^−1^)	51.6	54.5
Limit of quantification (ng spot^−1^)	152.2	165.8

The sensitivity of the method was determined by LOD and LOQ. Signal to noise ratio method was followed. S/N ratio was determined by spotting blank methanol. LOD and LOQ were judged as 3:1 and 10:1, respectively. The known concentrations of standards were diluted until their responses were three or ten times the standard deviation of the responses for blank. LOD and LOQ were found to be 51.6, 152.2 ng for lyoniside and 54.5, 165.8 for lyoniresinol, respectively (Table 1).

The precision was established by six replicates at three concentration levels (500, 1000 and 1500 ng per spot). Percentage relative standard deviation was calculated to estimate the precision. Intra-day and inter day precision were done by repeating the same assay six times on the same day and on four consecutive days. Precision data on the intra and inter-day variation are summarized in Table 2. Low values of % relative standard deviation indicate that the method is precise for the analysis.

**Table 2.** Intra- and inter-day precision for lyoniside and lyoniresinol.

	Lyoniside				Lyoniresinol			
	Inter-day precision		Intra-day precision		Inter-day precision		Intra-day precision	
Concentration (ng spot^−1^)	Mean peak area±	% RSD	Mean peak area±	% RSD	Mean peak area±	% RSD	Mean peak area±	% RSD
500	3210.9 ± 34.11	1.06	3250.7 ± 46.99	1.44	3238.1 ± 35.27	1.089	3299.6 ± 56.68	1.71
1000	6204.23 ± 36.36	0.58	6412.15 ± 62.8	0.97	6442.19 ± 84.29	1.3	6385.4 ± 69.82	1.08
1500	10254.7 ± 90.92	0.88	10214.7 ± 63.73	0.62	10210.37 ± 81.99	0.8	10151.6 ± 117.6	1.15

The robustness of the method was evaluated by determining the effects of small changes in method variables, i.e. variation in the composition of mobile phase or wavelength for detection. The mobile phase selected for the analysis was toluene: ethyl acetate: formic acid (4:3:0.4). Formic acid content was kept constant and toluene and ethyl acetate content were changed for a constant volume output. It yielded two form of solvent system one non polar (4.5:2.5:0.4) and another polar (3.5:3.5:0.4). Corresponding peak areas and % relative standard deviation were calculated to estimate the deviation from original method. Percentage relative standard deviation values between 1.01–1.25 and 0.94–1.27 showed that small deliberate change in solvent system does not greatly affect the analysis (Table 3).

**Table 3.** Robustness studies for lyoniside and lyoniresinol.

Change in mobile phase			Mean area ± SD		% RSD	
Actual	Used	Level	LS	LR	LS	LR
4:3:0.4	3.5:3.5:0.4	−5	5995.9 ± 71.71	6339.46 ± 81.07	1.19	1.27
	4:3:0.4	0	6120.7 ± 62.07	6425.76 ± 60.96	1.01	0.94
	4.5:2.5:0.4	+5	6275.8 ± 78.45	6261.76 ± 72.53	1.25	1.15
Wavelength change						
254	250	−4	6439.5 ± 60.71	6337.03 ± 68.46	0.94	1.08
	254	0	6357.8 ± 61.96	6432.36 ± 65.55	0.97	1.01
	258	+4	6441.8 ± 67.11	6268.03 ± 66.15	1.04	1.05

Another variable for detecting the robustness of method was detection wavelength. The analysis was done at 254 nm so for robustness analysis, ±4 nm change was deliberated in wavelength. The resultant % relative standard deviation was between 0.94–1.04 and 1.01–1.08, respectively, for lyoniside and lyoniresinol (Table 3).

The specificity of the method was established by analyzing test samples and reference standards. The position and purity of the spots for lyoniside and lyoniresinol in sample were substantiated by comparing R_f_ values and UV spectra of spots, respectively.

The accuracy of the developed system was ascertained by determining the recovery of the content being used to spike the pre-analyzed samples. The samples were spiked with 50%, 100% and 150% of the standard lyoniside and lyoniresinol, respectively and reanalyzed. The content of both components were quantified and percentage recovery was calculated. 99.73–100.2% lyoniside content was recovered while 99.46–100.4% lyoniresinol content was recovered. The results for the recovery study are depicted in Table 4.

**Table 4.** Accuracy as recovery studies for lyoniside and lyoniresinol.

% of reference standard used for spiking	Theoretical content	Amount of drug recovered	% of drug recovered	% RSD
Lyoniside				
0	2.5	2.49 ± 0.015	99.6	0.61
50	3.75	3.74 ± 0.02	99.73	0.53
100	5	5.01 ± 0.01	100.2	0.3
150	6.25	6.24 ± 0.03	99.84	0.48
Lyoniresinol				
0	2.5	2.501 ± 0.011	100.04	0.46
50	3.75	3.73 ± 0.015	99.46	0.4
100	5	4.99 ± 0.03	99.8	0.64
150	6.25	6.23 ± 0.02	99.68	0.23

### Quantification

**Figure 5.**
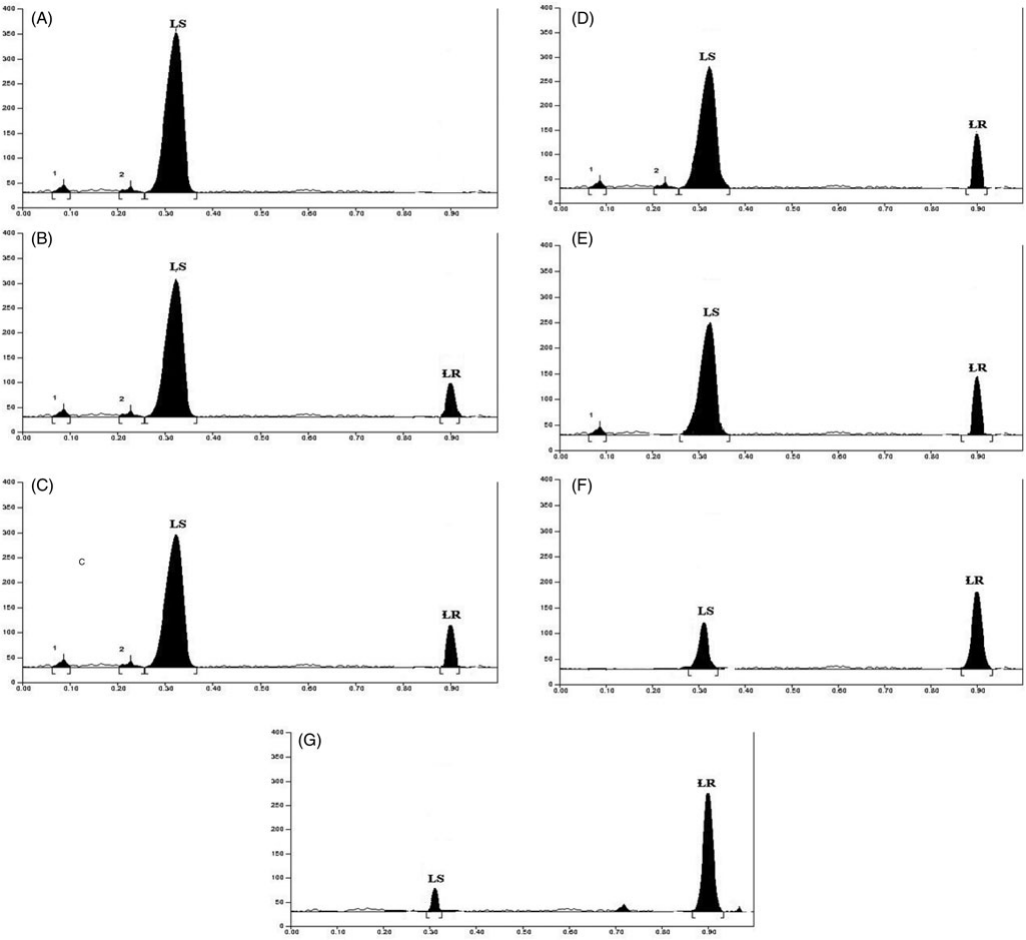
HPTLC chromatograms for aqueous extract and biotransformed extract (2–8 days).

The process of biotransformation was evaluated in terms of changes in lyoniside and lyoniresinol concentration over a period of time. As is evident from Figure 5, the contents of lyoniside and lyoniresinol went under a major change. As the time progresses, the content of lyoniside decreases whereas content of lyoniresinol increases (Table 5).

**Table 5.** Quantification of lyoniside and lyoniresinol in aqueous extract and biotransformed product.

No. of days	LS	LR
0	13.98 ± 0.4	3.42 ± 0.06
1	13.54 ± 0.2	3.4 ± 0.04
2	13.44 ± 0.3	3.25 ± 0.03
3	12.2 ± 0.5	4.6 ± 0.02
4	10.3 ± 0.1	6.1 ± 0.05
5	6.4 ± 0.2	9.2 ± 0.02
6	5.3 ± 0.5	11.1 ± 0.04
7	4.3 ± 0.2	12.4 ± 0.02
8	3.7 ± 0.4	13.2 ± 0.03

## Conclusion

The present work gives an account of change in chemical constituent of extract during fermentation using *W. fruticosa*. Lignan glycoside, lyoniside successfully transformed into lyoniresinol during this process. A new HPTLC method was developed and validated for their quantification. The developed method was unique as it simultaneously quantifies glycoside and aglycone.
